# Dental photon-counting computed tomography for the assessment of peri-implant structures

**DOI:** 10.1186/s40729-025-00640-8

**Published:** 2025-08-09

**Authors:** Maurice Ruetters, Christian Mertens, Holger Gehrig, Sinan Sen, Ti-Sun Kim, Hans-Peter Schlemmer, Stefan Schoenberg, Matthias Froelich, Marc Kachelrieß, Stefan Sawall

**Affiliations:** 1 Department of Operative Dentistry, Heidelberg University, University Hospital Heidelberg, Im Neuenheimer Feld 400, 69120 Heidelberg, Germany; 2 Department of Oral- and Maxillofacial Surgery, Heidelberg University, University Hospital Heidelberg, Im Neuenheimer Feld 400, 69120 Heidelberg, Germany; 3 Department of Orthodontics, University Hospital of Schleswig-Holstein, Arnold- Heller-Straße 3, 24105 Kiel, Germany; 4 Department of Radiology, German Cancer Research Center (DKFZ), Im Neuenheimer Feld 280, 69120 Heidelberg, Germany; 5 Department of Clinical Radiology and Nuclear Medicine, University Hospital Mannheim, Theodor-Kurz-Ufer 1-3, 68167 Mannheim, Germany; 6 Medical Faculty, Heidelberg University, Im Neuenheimer Feld 672, 69120 Heidelberg, Germany; 7 Division of X-Ray Imaging and CT, German Cancer Research Center (DKFZ), Im Neuenheimer Feld 280, 69120 Heidelberg, Germany

**Keywords:** Peri-implant bone, Photon-counting CT, CBCT, Metal artifacts, 3D dental imaging, Implant diagnostics, Image quality

## Abstract

**Purpose:**

To assess the diagnostic performance of photon-counting computed tomography (PCCT) in the imaging of peri-implant bone structures and to compare it quantitatively and qualitatively to cone-beam computed tomography (CBCT).

**Methods:**

Thirty titanium implants were placed in ten porcine mandibles. CBCT and PCCT scans were acquired and compared quantitatively regarding image noise and CT-values. Additionally bone thickness was compared to a gold standard at 60 standardized locations by one calibrated investigator in both modalities. Measurement accuracy was assessed by Bland–Altman analysis. Two experienced raters performed qualitative assessments of anatomic structures around the implant using a 5-point visibility scale. These included the bone-implant interface around the implant surface, the bone at the implant shoulder as well as the oral and vestibular bone lamella. Inter-rater agreement was assessed using ICC.

**Results:**

Across all evaluated implants, CT-values in a soft-tissue region of interest adjacent to the implant increased by 11.7 ± 3.9% for CBCT acquisitions, whereas they decreased by 5.3 ± 1.3% for PCCT acquisitions. Similarly, image noise in the respective ROIs is increased by a factor of 63 ± 13% in case of CBCT acquisitions and only by 23 ± 5% in case of PCCT acquisitions. Bone thickness deviations were smaller for PCCT (mean ± SD: 0.06 ± 0.08 mm) than for CBCT (0.39 ± 0.34 mm). Qualitative assessments consistently favored PCCT (*p* < 0.05) with excellent inter-rater reliability (ICC > 0.75 ) in almost all categories.

**Conclusions:**

PCCT enables superior visualization of peri-implant bone structures with fewer artifacts and improved diagnostic accuracy.

## Background

Photon-counting computed tomography (PCCT) represents a groundbreaking advancement in computed tomography technology, offering significant improvements in spatial resolution, soft tissue contrast, and radiation dose efficiency compared to conventional energy-integrating (EI) systems [1]. Particularly in dental imaging, PCCT presents promising applications. Previous studies have already investigated its suitability for visualizing teeth, their anatomical structures, surrounding tissues, and associated pathologies [2–4]. Furthermore, it has been demonstrated that PCCT surpasses the current standard, cone beam computed tomography (CBCT), both in terms of radiation dose efficacy and image quality [5].

A precise visualization of the osseointegration of implants and potential peri-implant changes such as bone loss or inflammation is essential for the long-term evaluation of implant success. While conventional two-dimensional (2D) imaging, such as panoramic radiographs (PA) or intraoral radiographs (IO), remains an important tool in dental diagnostics, they face considerable limitations, particularly in the assessment of peri-implant structures [6]. The 2D radiographic imaging techniques only provide superimposed projections of anatomical structures, making an accurate three-dimensional (3D) analysis of peri-implant bone impossible. Critical areas, such as the buccal and lingual bone plates, cannot be reliably assessed in 2D imaging due to superimposition with other structures or projection distortions [7].

Three-dimensional imaging using CBCT or conventional CT offers substantial advantages over 2D radiographic diagnostics by allowing for the reconstruction of sectional images in multiple planes, thereby enabling a more precise assessment of pathological conditions. However, despite its advantages, CBCT has notable limitations. One of its primary weaknesses is the restricted contrast resolution, making it difficult to differentiate between bone and implant material [8]. Another major issue with CBCT is the presence of metal artifacts. These artifacts frequently obscure, distort, or even entirely mask adjacent structures, complicating accurate diagnostics [8]. This limitation is particularly pronounced when evaluating fine bone structures, which are often present in buccal or lingual regions. Furthermore, quantitative image analysis with CBCT is highly restricted since the systems are often not calibrated to result in actual CT-values but show arbitrary units.

Photon-counting computed tomography (PCCT) addresses many of these limitations by providing superior spatial and contrast resolution, enhanced metal artifact reduction, and the ability for spectral imaging [9, 10]. PCCT detectors count individual X-ray photons and sort them based on their energy, enabling dual- or even multi-energy acquisitions with a single detector. Furthermore, to account for the high x-ray flux rates in clinical CT, the detector pixels are much smaller compared to conventional CT systems, allowing for a higher spatial resolution and hence, for example, enabling the visualization of fine anatomical structures while reducing radiation exposure at the same time [11–14]. Particularly in detecting early peri-implant pathologies such as bone resorption or peri-implantitis, PCCT could serve as a superior diagnostic modality. Another potential benefit of PCCT over CBCT is the fact that clinical PCCT systems are equipped with high-power x-rays sources that surpass the tubes usually found in CBCT systems. This in particular allows for the usage of patient specific prefilters, composed of tin (Sn) or silver (Ag) [15]. These prefilters increase the effective energy of the observed x-ray spectrum by absorbing low energy photons and consequently result in a reduction of metal induced blooming and artifacts.

Given these promising properties, the present study aims to evaluate whether photon-counting computed tomography is suitable for the detailed visualization of peri-implant structures. The hypothesis is that PCCT can depict peri-implant structures more accurately and with higher quality, both quantitatively and qualitatively compared to CBCT.

## Results

### Quantitative image analysis

Across all evaluated implants, CT-values in a soft-tissue region of interest adjacent to the implant increased by 11.7 ± 3.9% for CBCT acquisitions, whereas they decreased by 5.3 ± 1.3% for PCCT acquisitions. Specifically, CBCT acquisitions exhibited blooming artifacts that extended into the surrounding soft tissue and hence result in increased CT-values while PCCT acquisitions were characterized by a reduction in CT values more indicative of beam-hardening artifacts. Consequently, the magnitude of implant‐induced artifact in PCCT is approximately half that observed in CBCT. Similarly, image noise in the respective ROIs is increased by a factor of 63 ± 13% in case of CBCT acquisitions and only by 23 ± 5% in case of PCCT acquisitions.

**Fig. 1:**
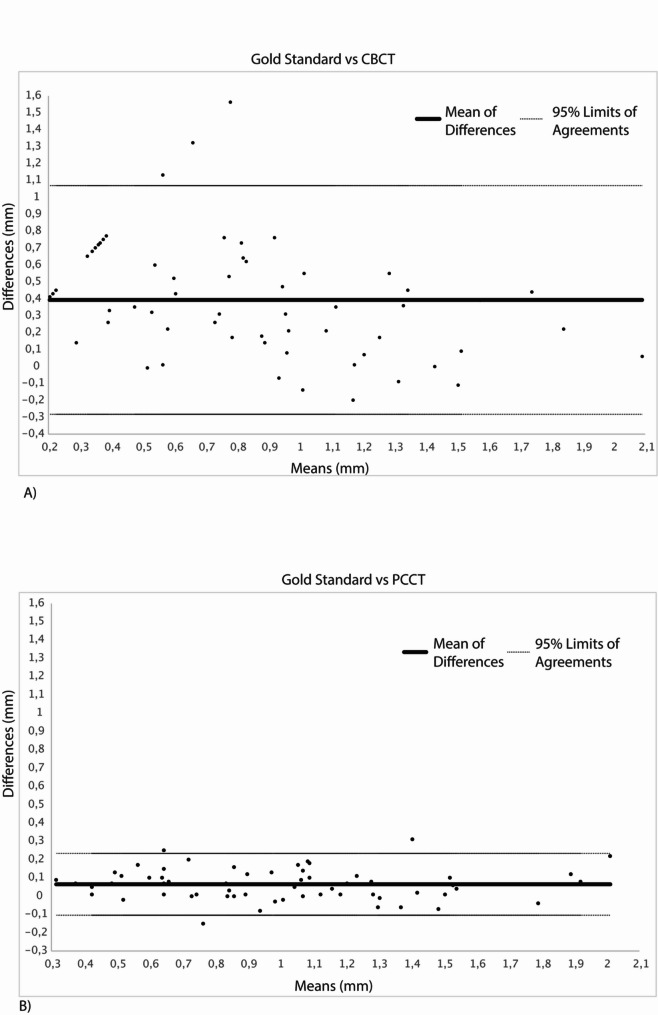
Bland altman plots: Gold-standard compared to CBCT (left) and gold standard compared to PCCT (right). The partially linear pattern of data points on the left side of the CBCT Bland–Altman plot can be attributed to the absence of measurable bone at these locations in the CBCT scans

The agreement between the bone thickness (bt) measurements from the imaging modalities and the reference standard was evaluated using Bland-Altman analysis. The corresponding results are presented in two Bland-Altman plots (Fig. 1), showing the mean differences and the 95% limits of agreement for each modality in comparison to the reference. For CBCT measurements, the mean difference relative to the reference was 0.39 mm, with 95% limits of agreement ranging from − 0.28 mm to 1.06 mm. For PCCT measurements, the mean difference compared to the reference was 0.06 mm, with 95% limits of agreement from − 0.10 mm to 0.23 mm. These values are visualized in Fig. 1 and provide a quantitative basis for comparing the measurement agreement of both imaging modalities with the reference. At 14 out of the 60 locations analyzed, noo bone was visible in the CBCT, even though bone was present. This was not the case at any location in PCCT.

### Qualitative image analysis

**Fig. 2:**
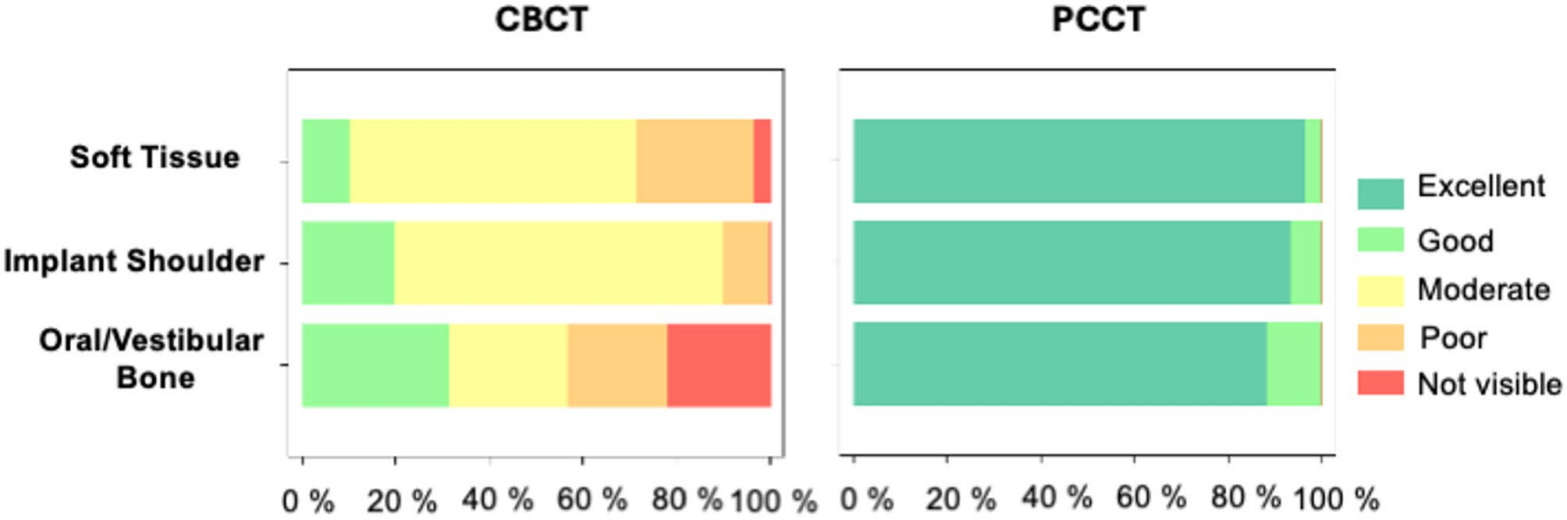
Results of the qualitative assessment. The visualization of relevant anatomical structures was evaluated by two experienced readers using a 5-point scale. As demonstrated in the Bland–Altman plots, there were several regions in the CBCT scans where the oral and vestibular cortical bone plates were not discernible due to artifacts. This limitation is also reflected in the proportion of sites rated as ‘not visible’ in the CBCT analysis (marked in red). In contrast, all corresponding sites were clearly visible in the PCCT images

The subjective image quality of the PCCT was consistently rated as superior when compared to the CBCT across all anatomical structures that were analyzed. Specifically, this applied to the assessment of the bone-implant interface, the evaluation of the bone at the implant shoulder, as well as the depiction of the vestibular and oral bone regions (Fig. 2). In general, all of these anatomical structures received ratings ranging from good to excellent in the PCCT acquisitions, whereas in the CBCT acquisitions, the ratings varied more widely—from not visible in some instances to excellent in others. This discrepancy highlights a clear advantage of PCCT in terms of consistent visualization quality. Importantly, the differences in image quality evaluations between PCCT and CBCT were statistically significant for each of the three anatomically distinct regions assessed, with a p-value of less than 0.05 (Fig. 2). These findings underscore the diagnostic benefit of PCCT over CBCT in this context. In terms of consistency between evaluators, the inter-rater correlation coefficient for the CBCT was classified as good for the oral and vestibular bone (0.649), and as excellent for the bone-implant interface (0.838) and the bone at the implant shoulder (0.766). In contrast, the PCCT demonstrated excellent inter-rater agreement across all evaluated anatomical regions, with coefficients of 1.000 for the bone-implant interface, 0.769 for the bone at the implant shoulder, and 0.843 for the oral and vestibular bone (Table 1). These results further support the robustness and reproducibility of image interpretation when using PCCT.

**Table 1:** ICC values for qualitative ratings

	PCCT	CBCT
**Bone-implant interface**	1	0.838
**Bone at Implant Shoulder**	0.769	0.766
**Oral/Vestibular Bone**	0.843	0.639

## Discussion

The present study demonstrates the superiority of PCCT over CBCT in the visualization of peri-implant tissues. This applies to both quantitative and qualitative evaluations. Thus, to our knowledge, the results of this study represent a key advancement in 3D imaging of peri-implant bone structures, which has not been possible in this way before.

A quantitative evaluation of image quality in terms of implant-induced changes of CT-values and noise revealed that the CBCT is more susceptible to distortions caused by the metal artifacts. In particular, CBCT acquisitions show blooming artifacts that range into the soft tissue while the PCCT acquisitions show a decrease in CT-values coherent with typical beam hardening artifacts. A subjective evaluation of image quality in terms of the delineation of structures such as surrounding implant bone in the vicinity of artifacts revealed that PCCT is consistently rated higher than the same regions acquired using CBCT. This holds for all investigated structures with statistical significance (all < 0.05). The high intra-class correlation coefficient between both raters with high ICC-values for all structures validates these findings and aligns with previously published data on PCCT for other dental structures (2, 5, 6).

The Bland–Altman plots illustrate the agreement between the gold standard and the two imaging modalities, CBCT (Figure A) and PCCT (Figure B), in measuring the thickness of the vestibular and oral cortical bone plates. In the plot comparing CBCT to the gold standard (Figure A), a wide dispersion of data points is evident, particularly in the lower range of mean values. A partially linear pattern appears on the left side of the plot, indicating that, in several cases, CBCT failed to detect measurable bone, likely due to blooming artifacts. The limits of agreement are broad, and the mean difference is shifted above zero, suggesting a tendency of CBCT to underestimate bone thickness in comparison to the gold standard. These findings point to both systematic measurement errors and limited precision in thinner bone regions. In contrast, the plot comparing PCCT to the gold standard (Figure B) shows a narrow distribution of data points closely clustered around the mean difference line. The mean difference is near zero, and the limits of agreement are substantially narrower than in the CBCT plot. This reflects a high degree of agreement and measurement accuracy between PCCT and the gold standard. No systematic deviation across the measurement range is observed, and only a few outliers are present. Overall, these results demonstrate and confirm the clear advantage of PCCT over CBCT in terms of detection, accuracy and reproducibility when assessing cortical bone thickness. PCCT provides more consistent and reliable measurements, particularly in anatomical regions where CBCT is limited by image artifacts or resolution constraints.

Notably, the ability of PCCT to reliably depict delicate buccal and oral bone structures surrounding implants represents a groundbreaking advancement in implant imaging. This enhanced visualization could enable a more precise detection of peri-implant defects compared to CBCT. Consequently, clinicians may benefit from improved prognostic assessments and more accurate treatment planning.

This study has several limitations. The porcine jaws used in this study do not fully replicate a human clinical scenario, as they lack the surrounding human soft and hard tissue anatomy such typically present in a real patient. Consequently, the observed intersection lengths are generally shorter than those found in actual patient scans, and image noise would likely be more pronounced in a clinical setting compared to the results obtained here. However, this limitation applies equally to both imaging modalities, ensuring the validity of the comparisons made. It can be assumed that, in real-world clinical settings, the difference in image quality between the two modalities would be even more pronounced. One possible reason is the variation in patient positioning during image acquisition. CBCT scans are typically performed with the patient in a standing or seated position, which increases the likelihood of motion artifacts. In contrast, PCCT imaging is conducted with the patient in a supine position, minimizing movement-related distortion [8]. Additionally, scan duration differs significantly between the two modalities. In the present study, the radiation time for CBCT was 14.2 s, whereas for PCCT it was only 3 s but could be further improved to less than a second, given the dual-source nature of the Naeotom Alpha system. The longer scan duration associated with CBCT further increases the risk of patient movement and associated artifacts [8]. This study was conducted exclusively using titanium implants. Therefore, the results cannot be directly extrapolated to other implant materials, such as ceramic implants. Further studies are required to evaluate the applicability of these findings in such contexts. Previous research involving CBCT has already demonstrated that zirconia implants tend to produce more pronounced artifacts compared to titanium implants, which could further influence measurement accuracy [16].

## Conclusions

The hypothesis of this study - that PCCT can depict peri-implant structures more accurately and with higher quality, both quantitatively and qualitatively compared to CBCT - was confirmed. The results of this study could mark a new era in 3D visualization of peri-implant hard tissue. In the future, it could potentially replace CBCT, offering reliable three-dimensional imaging of peri-implant bone while reducing radiation exposure. However, further preclinical and clinical studies are necessary, along with the widespread implementation of PCCT in routine clinical practice.

## Materials and methods

### Study protocol

**Fig. 3:**
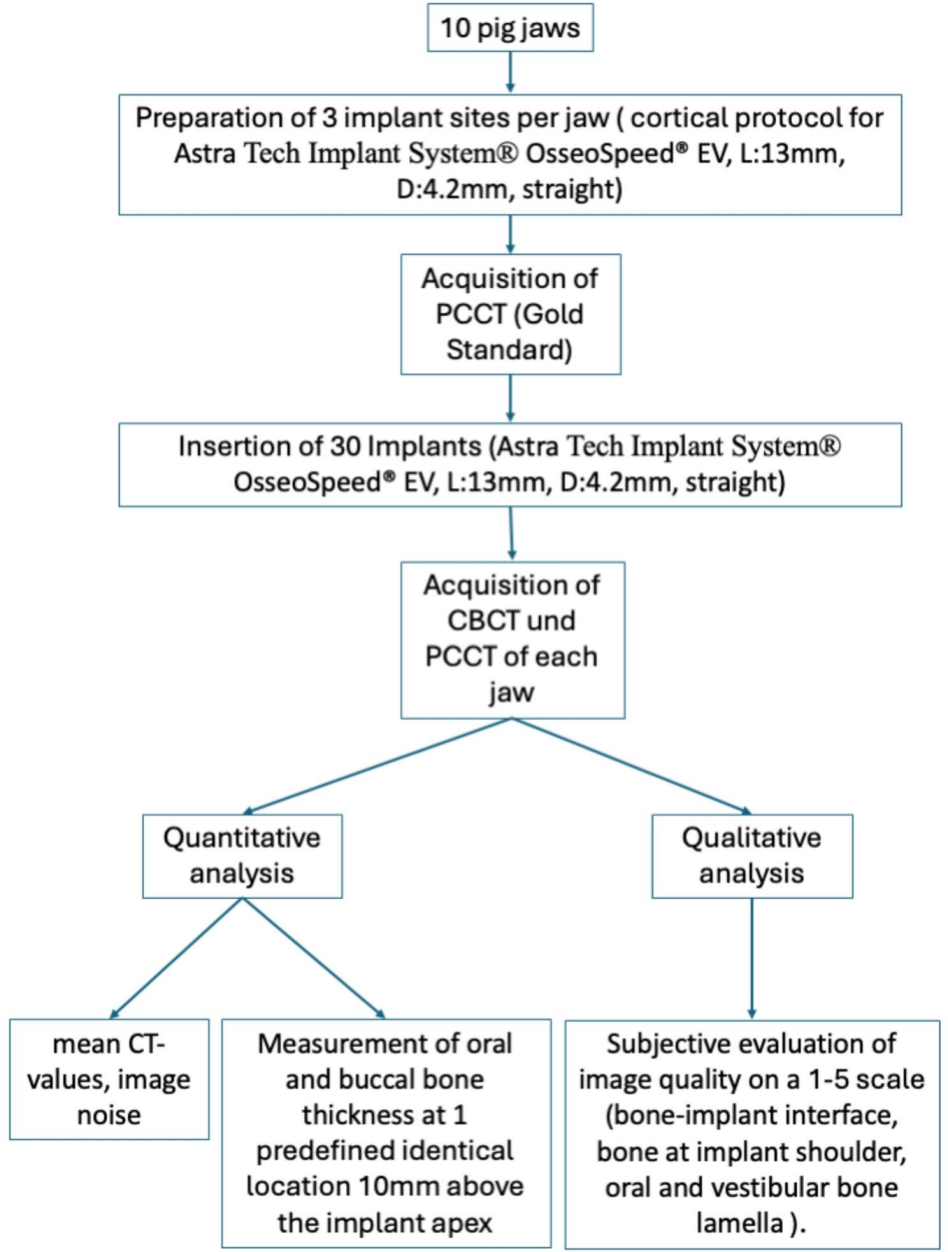
Flow-chart of the study

In total, the adjacent vestibular peri-implant bone tissues surrounding 30 titanium implants are examined. These implants are placed in the mandibular angle of 10 porcine mandibles. A distance of 15 mm is left between each implant. Straight Astra Tech Implant System^®^ OsseoSpeed^®^ EV implants (DentsplySirona, Bensheim, Germany) with a diameter of 4.2 mm and a length of 13 mm, are utilized in this study [17]. After reflection of the gingiva, site preparation is carried out following the manufacturer’s Cortical protocol. At the coronal aspect, the osteotomy corresponds precisely to the implant diameter, whereas the medial portion is underprepared by 0.15 mm, and the apical portion by 0.5 mm [17]. Subsequently, PCCT scans (Naeotom Alpha, Siemens Healthineers, Forchheim, Germany) of the implant beds are acquired. These images serve as the gold standard and reference for the quantitative image analysis, described later. Following this, the implants (Astra Tech Implant System^®^ OsseoSpeed^®^ EV, straight, Diameter 4.2 mm, Length 13 mm, DentsplySirona, Bensheim, Germany) are inserted precisely at the crestal level. The soft tissue is then sutured at the wound margins, ensuring complete coverage of the implants. Subsequently, a CBCT scan is performed, followed by PCCT scan of the mandibles. The workflow is illustrated in Fig. 3.

### CT systems, image acquisition and reconstruction

The CBCT system used in this study (Orthophos SL 3D, Dentsply Sirona, Bensheim, Germany) is operated in high-resolution mode using a tube voltage of 85 kV, a tube current of 6 mA, a scan time of 14.2 s and a field of view of 8 × 8 cm^2^ resulting in a dose-area product of 0.94 Gy·cm². Image reconstruction is performed using filtered backprojection onto an appropriately sized matrix with an isotropic voxel size of 160 μm [10]. A summary of all acquisition and reconstruction parameters can be found in Table 2. The clinical photon-counting CT system (Naeotom Alpha, Siemens Healthineers, Forchheim, Germany) used in this study is a dual-source CT system and provides a field of measurement (FOM) of 50 cm. The photon-counting detector used in each imaging chain can be operated in several distinct modes. A mode referred to as ultrahigh resolution (UHR) is of particular interest herein. Using this mode, the system provides a detector pixel size of 150 μm × 170 μm in the center of rotation and allows for a spatial resolution of up to 42.9 lp/cm (MTF_10%_) [11]. All acquisitions presented herein have been performed with the detector operated in said UHR mode and using a tube voltage of 140 kV and using an additional 0.4 mm tin prefilter. The tube current was chosen to result in a dose similar to the one observed in CBCT acquisitions. Image reconstruction was performed with an axial voxel size of 160 μm, a slice thickness of 200 μm and a slice increment of 100 μm using a sharp B72 kernel using the default reconstruction method of the system, i.e., Quantum Iterative Reconstruction (QIR) at a strength of 3 [12, 13].

**Table 2:** Summary of all scan and reconstruction parameters for the experiments presented herein. (FBP-filtered backprojection, QIR3-quantum iterative reconstruction with strength 3)

	CBCT	PCCT
Dose	0.94 Gy·cm² (DAP)	4 mGy (CTDI_16 cm_)
Tube Voltage	85 kV	140 kV
Additional Prefilter	-	0.4 mm Sn
Trajectory	Circle	Spiral
Reconstruction	FBP	QIR3
Voxel Size	160 μm	160 μm
Slice Thickness	160 μm	200 μm
Slice Increment	160 μm	100 μm
Scan time	14.2 s	3.0 s

### Quantitative image analysis

To quantitatively assess image quality, mean CT-values and image noise (standard deviation) of the CT-values in the peri-implant tissue were measured as a surrogate parameter for the extent of metal artifacts originating from the implants similar to methods described in the literature [18]. In particular, we evaluate the relative change of CT-values and image noise in a soft tissue region-of-interest between acquisitions with and without implants. Given that the reconstructions of each mandible are registered to one another, this approach allows for the quantification of potential implant induced image quality degradations.

**Fig. 4:**
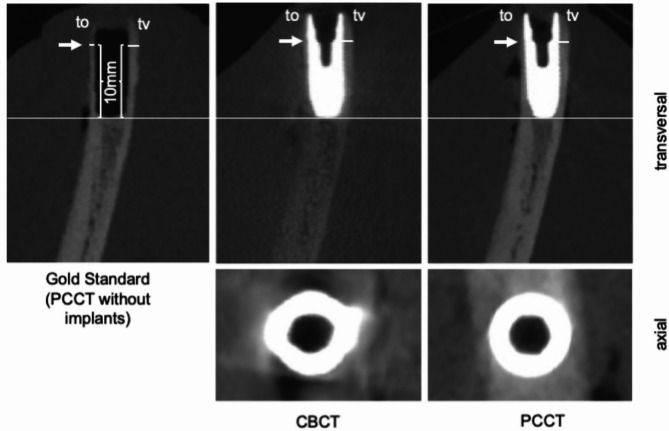
Representation of the measurement systematics and the different imaging techniques in PCCT and CBCT. The images are registered to one another. The subjectively better image quality of the PCCT scans is clearly visible. Furthermore, the oral bone lamella (to) can be identified in both the gold standard and the PCCT images with implants at the same location (indicated by the arrow). In contrast, in the CBCT image, this is not assessable or visible at the same location due to the artifacts generated. This is also visible in the axial slices. The lower level of artifacts in the PCCT also allows for the visualization of individual implant threads, as well as precise measurement of the vestibular bone thickness (tv)

Furthermore, the vestibular and oral bone thickness was determined at identical locations, each 10 mm away from the apical part of the osteotomy (Fig. 4). These sites were selected because, in the drilling protocol applied, the diameter of the implant site preparation matches the implant diameter over the coronal 3 mm, while the more apical portion remains underprepared. This technique ensures that the bone thickness, measured preoperatively via PCCT and serving as the reference standard, is maintained during implant placement and not compromised by the preparation process. Otherwise, a reliable comparison of bone thickness before and after implant insertion would not be feasible. The software OSIRIX pro (aycanOsiriX 2.06.000, aycan Digitalsysteme GmbH, Würzburg, Germany) was used. In total, bone thickness was determined at 60 identical locations in each of the three protocols. The thickness measurements at the implants in CBCT and PCCT were conducted by a calibrated examiner (MR), blinded to the gold standard and the procedure. The calibration was carried out by having the examiner perform 40 measurements on image datasets according to the specified protocol, which were not part of this study. These measurements were discussed with an experienced examiner, and any differences in opinion were resolved through consensus. The gold standard measurements in PCCT without implants were performed by experienced examiners in consensus (MR and HG). To eliminate memory effects, the gold standard measurements were performed one month after the initial assessments.

### Qualitative image analysis

Image quality of CBCT and PCCT with implants was independently assessed by two dentists (MR, HG), each of them with more than 10 years of experience. For the qualitative assessment of the images, two raters evaluated the visibility of diagnostically relevant structures. These included the bone-implant interface around the whole implant surface, the bone at implant shoulder as well as the oral and vestibular bone lamella. The evaluation was performed using a 5-point scale (1 = excellent, 2 = good, 3 = moderate, 4 = poor, 5 = not visible) like previous investigations, as previously applied in prior studies [5]. Investigators were blinded to each other and to the imaging modality (CBCT/PCCT).

### Statistical evaluation

Statistical analysis was performed using the R software package (version 4.3.1., R Foundation of Statistical Computing, Vienna, Austria). Continuous variables are expressed as mean ± standard deviation. Furthermore, inter-reader reproducibility was assessed using the intraclass correlation coefficient (ICC). The ICC is considered poor for ICC < 0.40, moderate to good for 0.40 < ICC < 0.75 and excellent for ICC > 0.75. Bland–Altmann plots were created including 95% limits of agreement where the measurements of PCCT without implants were defined as gold standard to detect possible over- or underestimation and systematic bias. The paired Wilcoxon signed rank test with continuity correction was used to compare qualitative imaging scores. A p-value below 0.05 is considered statistically significant.

## Data Availability

No datasets were generated or analysed during the current study.

## Abbreviations

CBCT: Cone beam computed tomography
CT: Computed tomography
CTDI: CT dose index
FOV: Field of view
ICC: Intra-class correlation coefficient
PCCT: Photon-counting CT
QIR: Quantum Iterative Reconstruction
SD: Standard deviation
